# Analysis of the Spatial Distribution of New Cases of Leprosy in the State of Pernambuco, Northeast Brazil

**DOI:** 10.3390/tropicalmed10120327

**Published:** 2025-11-21

**Authors:** Celivane Cavalcanti Barbosa, Gilberto Silva Nunes Bezerra, Amanda Tavares Xavier, André Luiz Sá de Oliveira, Marcílio Sandro de Medeiros, Cristine Vieira do Bonfim, Zulma Maria de Medeiros, Wayner Vieira de Souza

**Affiliations:** 1 Department of Collective Health, Aggeu Magalhães Institute, Oswaldo Cruz Foundation, Recife 50740-465, Brazil; 2 Department of Life & Health Sciences, Dundalk Institute of Technology, A91 K584 Dundalk, Ireland; gilberto.bezerra@dkit.ie; 3 Nursing Department, Academic Center of Vitória-CAV, Federal University of Pernambuco, Vitória de Santo Antão 55608-680, Brazil; amanda.txavier@gmail.com; 4 Center for Statistics and Geoprocessing, Aggeu Magalhães Institute, Oswaldo Cruz Foundation, Recife 50740-465, Brazil; andre-luiz.oliveira@fiocruz.br; 5 Department of Studies on Violence and Health Jorge Careli, National School of Public Health Sergio Arouca, Oswaldo Cruz Foundation (Fiocruz), Rio de Janeiro 21040-361, Brazil; marcilio.medeiros@fiocruz.br; 6 Social Research Division, Joaquim Nabuco Foundation, Ministry of Education, Recife 52171-010, Brazil; cristine.bonfim@uol.com.br; 7 Department of Parasitology, Aggeu Magalhães Institute, Oswaldo Cruz Foundation, Recife 50740-465, Brazil; zulma.medeiros@fiocruz.br; 8 Graduate Program in Health Sciences, University of Pernambuco, Recife 50100-130, Brazil

**Keywords:** leprosy, neglected diseases, ecological studies, spatial analysis, public health

## Abstract

Spatial analysis of leprosy case distribution serves as a critical tool for identifying priority areas for intervention, particularly in settings with marked epidemiological heterogeneity. This study aimed to analyze the spatial distribution of new leprosy cases in Pernambuco, Brazil, 2000–2024. This is an ecological study with the municipalities of residence as the units of analysis. The data was extracted from the Notifiable Diseases Information System. The average incidence rates were calculated: general, in children under 15 years of age and with grade 2 physical disability at diagnosis, for four five-year periods. Bayesian smoothing and Moran’s global and local autocorrelation statistics were applied. The average rates of overall detection were 29.0/100,000 inhabitants per year (very high); in children under 15 years of age, 10.7/100,000 inhabitants per year (hyperendemic); and grade 2 physical disability, 1.6/100,000 inhabitants per year (low). Spatial analysis showed significant spatial heterogeneity, with clusters of high overall detection rates of leprosy cases, high detection rates among children under 15, and high rates of grade 2 physical disability at diagnosis, mainly in macro-regions I and IV. There is circulation of *Mycobacterium leprae* across all macro-regions of the state, with evidence of active transmission foci in macro-region III. Therefore, municipalities with a priority for intervention are concentrated in macro-regions I and IV, highlighting the need to strengthen leprosy surveillance and control actions in the state.

## 1. Introduction

Leprosy is a neglected infectious disease that may lead to visible deformities and physical disabilities, associated with substantial socio-economic and psychological burdens [1,2,3]. The etiological agents are *Mycobacterium lepromatosis* and *Mycobacterium leprae*, obligate intracellular bacteria with an affinity for the peripheral nervous system [4]. The incubation period for the disease is on average five years, ranging from two to seven years [4,5]. The late diagnosis of cases contributes to transmission and can lead to the evolution to more serious clinical forms [6].

After the introduction of polychemotherapy in the 1980s, there was a worldwide decline in the prevalence of leprosy; however, eliminating the disease is still a challenge [7]. In 2023, 182,815 new cases were recorded worldwide [8]. Brazil was responsible for 12.5% (22,773) of these cases, ranking second in absolute number and contributing 91.9% of new diagnoses in the Americas [8].

Brazil has sought to achieve the global targets for leprosy control [9,10]. However, these advances have been impacted by socioeconomic and environmental factors [11]. In response to these challenges, the Ministry of Health has launched a new National Strategy to Combat Leprosy 2024–2030 [12].

One of the fundamental pillars of the new strategy is the maintenance of active epidemiological surveillance to support decisions [12]. This involves the collection, processing, analysis and continuous interpretation of data related to leprosy cases and their contacts [13,14,15]. Active surveillance significantly increases the identification of never-before-treated leprosy cases in low-endemic areas, highlighting the importance of tracing household and neighbor contacts [14].

In order to deepen knowledge about leprosy, especially in relation to its surveillance, spatial analysis can be used. This technique makes it possible to identify priority areas for health interventions [16,17]. In research on leprosy using this approach, clusters of high risk of disease transmission were identified [7]. Other studies have verified the heterogeneity of the distribution of cases, highlighting areas of hyperendemicity [18,19]. These studies highlight the importance of using spatial analysis to understand and combat leprosy as a public health problem in Brazil [20,21].

The state of Pernambuco stood out with the highest number of leprosy cases in the Northeast of Brazil in 2019 and 2021 [22]. Despite the actions implemented, such as the Action Plan to Combat Neglected Diseases (Sanar), leprosy still persists in several regions of Pernambuco [23,24]. This scenario may be due to the heterogeneity of the area of the state, to the presence of an active chain of transmission and to the late diagnosis of the disease [21,23,25].

Thus, there is a need to better understand the course of leprosy in the state of Pernambuco, in northeastern Brazil. Once the priority areas for health interventions have been identified, this can subsidize the planning and decision making of local health authorities, promoting actions for early diagnosis and treatment. Therefore, this study aims to analyze the spatial distribution of new leprosy cases in Pernambuco between 2000 and 2024.

## 2. Materials and Methods

### 2.1. Area of Study

The study was conducted in the state of Pernambuco, in the northeast of Brazil. The state is composed of 184 municipalities and one state district (Fernando de Noronha Archipelago) [26], located 545 km from Recife, the state capital. The district was excluded from this analysis due to its isolated geographical location, which results in territorial discontinuity in relation to the other municipalities. This characteristic compromises the application of spatial analysis techniques based on correlation between neighboring municipalities.

**Figure 1:**
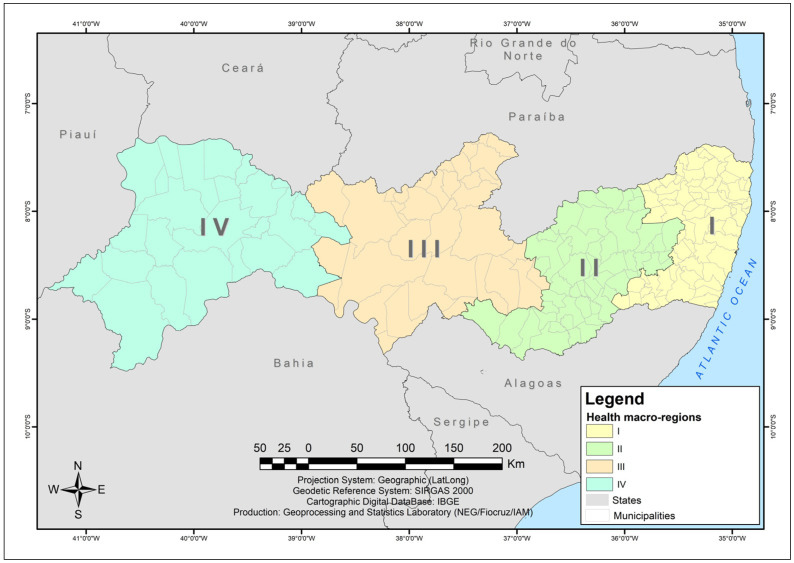
Location map of Pernambuco and health macro-regions. Source: Instituto Aggeu Magalhães (2024) [33].

In this research, the health macro-regions were adopted according to the regional health master plan, to help locate clusters of interventions: (I) Metropolitana; (II) Agreste; (III) Sertão; (IV) Vale do São Francisco (the valley of São Francisco River) and Araripe [27] (Figure 1). Health macro-regions are groupings of health regions (set of neighboring municipalities) defined politically and administratively by the Pernambuco state health department, based on cultural, economic and social similarities [28,29]. The regionalization of health allows for the decentralization of actions and services in an organized, supportive, hierarchical and resolutive manner [30,31,32]. The sociodemographic and economic characterization of the health macro-regions is detailed in Table 1.

### 2.2. Study Design

This is an ecological study whose unit of analysis was the municipalities. In order to present the results and discussion, the grouped municipalities were located within their respective macro-regions.

### 2.3. Period of Reference and Study Population

The study period covered 1 January 2000 to 31 December 2024, totaling 25 years of analysis. The starting year, 2000, was chosen because it was two years after decree no. 73/1998 [35], which regulated the use of the SINAN database (Notifiable Diseases Information System—Sistema de Informação de Agravos de Notificação) and made regular database entry mandatory. The final year, 2024, was chosen because it was the latest available database.

Notified cases were selected from the leprosy database according to the following criteria:

(a) Inclusion criteria: All new cases (individuals who have never received any specific treatment for the disease), new cases in children under the age of 15, new cases with grade 2 disability at the time of diagnosis, residents of Pernambuco, with a year of diagnosis within the study period;
(b) Exclusion criteria: All cases with a diagnostic error outcome.

### 2.4. Data Source

The data extracted from SINAN database was obtained from the compulsory notification forms and the leprosy monitoring bulletin. Population data for the municipalities and the digital cartographic grid of Pernambuco, in shapefile format, was obtained from the Brazilian Institute of Geography and Statistics (IBGE) [26,36]. Leprosy diagnosis in Brazil follows the guidelines of the Ministry of Health, which recommend dermatological and neurological examinations to detect skin lesions with altered sensitivity and/or peripheral nerve involvement [12].

An evaluation of duplicates, which are identical cases, was conducted on the SINAN database. The criteria adopted for identification included the full name of the person treated, date of birth, mother’s name, as well as the dates of notification, diagnosis and beginning of treatment. Subsequently, inconsistencies were assessed, i.e., consistency between variables. The analysis focused on the fields: operational classification, clinical form, dates of diagnosis and start of treatment. Only cases validated as new were kept.

### 2.5. Definition of Indicators

For the spatial analysis, three epidemiological indicators were chosen based on the epidemiological bulletin of the health surveillance secretariat of the Ministry of Health (2023) [37] (Table 2). The 25-year period was divided into four five-year periods: 2000 to 2004, 2005 to 2009, 2010 to 2014, 2015 to 2019 and 2020 to 2024. Incidence rates were calculated using the average for each five-year period per municipality. In the numerator, the total number of leprosy cases over the five years was added up and divided by five. In the denominator, the population in the middle of each five-year period was used, estimated based on data from 2002, 2007, 2012, 2017 and 2022, corresponding to each respective five-year period. Finally, the rates were multiplied by a factor of 100,000 to express them in standardized terms.

### 2.6. Data Analysis

The indicators per municipality were spatially distributed and categorized according to the parameters shown in Table 2. The local empirical Bayesian model was then applied to reduce the instability caused by the random variation in rates. This model is useful because it calculates a weighted rate, considering area variances, which helps to smooth fluctuations and improve the accuracy of estimates [39,40].

Bayes’ theorem is used to estimate the probability of an event occurring over time, providing a robust approach to dealing with data that can vary unpredictably [39,41]. The parameters of the epidemiological indicators shown in Table 2 were used in the study.

Thereafter, both Global and Local Moran’s I spatial statistics were calculated using the overall average incidence rates aggregated by municipality for each quinquennial period. The Global Moran’s Index (Moran’s I) ranges from −1 to +1 and reflects the degree of autocorrelation in the data set, based on the product of the deviations from the global average [42]. Values close to −1 indicate negative autocorrelation; values close to +1, positive autocorrelation; and values at 0, absence of autocorrelation [42].

Local autocorrelation was then performed, which involved three stages. The first stage was the identification of critical and transition areas using Box Map and Moran’s scatter diagram, which divides the data into four quadrants (Q): Q1—High/High (positive values and positive averages), Q2—Low/Low (negative values and negative averages), Q3—High/Low (positive values and negative averages) and Q4—Low/High (negative values and positive averages) [41].

In the second stage, the Local Indicator of Spatial Association (Lisa) was used, which allows the identification of regions with significance of 95, 99 and 99.9% (Lisa Map), as well as non-significant regions [40,41]. In the final stage, the Moran Map was generated, combining the areas with a positive spatial relationship identified in the Box Map and with spatial significance above 95% in the Lisa Map (5% significance level) [41]. The data was presented using the Box Map and the Moran Map, highlighting the area with priority for intervention, classified as High/Highest (Q1).

The spatial analysis calculations were performed using Terra View software, version 4.2 (National Institute for Space Research-INPE, São Paulo, Brazil). QGIS software, version 2.14 (Open Source Geospatial Foundation, Chicago, IL, USA), was used to present the cartographic data and create thematic maps.

## 3. Results

In the evaluation of the database of new leprosy cases, records that did not meet the eligibility criteria were excluded. Of the total of 65,338 new cases, 679 (1.0%) were excluded due to misdiagnosis. Duplication and inconsistency assessments were then carried out, identifying 137 (0.2%) duplicate cases and 26 (0.1%) cases of inconsistencies. After these steps, 64,496 (98.7%) new cases were considered eligible for this study.

Of the 64,496 eligible cases, 6156 (9.5%) occurred in children under 15, and 3535 (5.5%) had grade 2 physical disability at the time of diagnosis. During the 25-year period analyzed, the average leprosy detection rates were as follows: new cases, 29.0 per 100,000 inhabitants per year (very high classification); cases in children under 15, 10.7 per 100,000 inhabitants per year (hyperendemic); and cases with grade 2 physical disability at the time of diagnosis, 1.6 per 100,000 inhabitants per year (low classification).

In the spatial analysis of the three epidemiological indicators selected, spatial autocorrelation was observed in all five-year periods, as indicated by the global Moran index (Moran’s I) (Table 3).

### 3.1. Spatial Analysis of General Incidence

**Figure 2:**
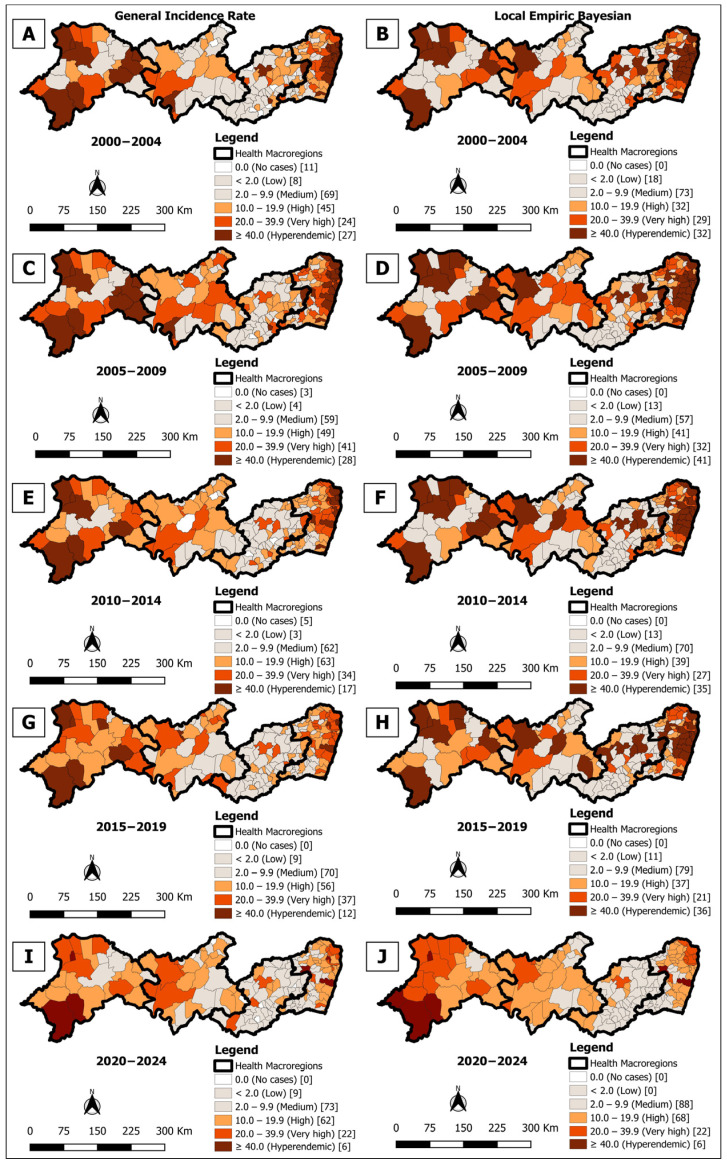
Spatial analysis on the general incidence rate of leprosy for incidence rate of leprosy (**A**,**C**,**E**,**G**,**I**) and rate smoothed using the local empirical Bayes method (**B**,**D**,**F**,**H**,**J**), per 100,000 inhabitants, according to the municipality. Pernambuco, Brazil, over five-year period (2000–2024).

In the analysis of the five quinquennia (2000–2024), a progressive reduction was observed in the number of municipalities classified as hyperendemic by crude detection rate, from 27 (14.7%) in 2000–2004 to six (3.3%) in 2020–2024 (Figure 2A,C,E,G,I). However, Bayesian smoothing identified additional municipalities with hyperendemicity in all periods, revealing areas of hidden endemicity: 5 new records in 2000–2004 (Figure 2B), 13 in 2005–2009 (Figure 2D), 18 in 2010–2014 (Figure 2F), and 24 in 2015–2019 (Figure 2H), with the exception of the quinquennium 2020–2024 (Figure 2J), which remained with six municipalities.

**Figure 3:**
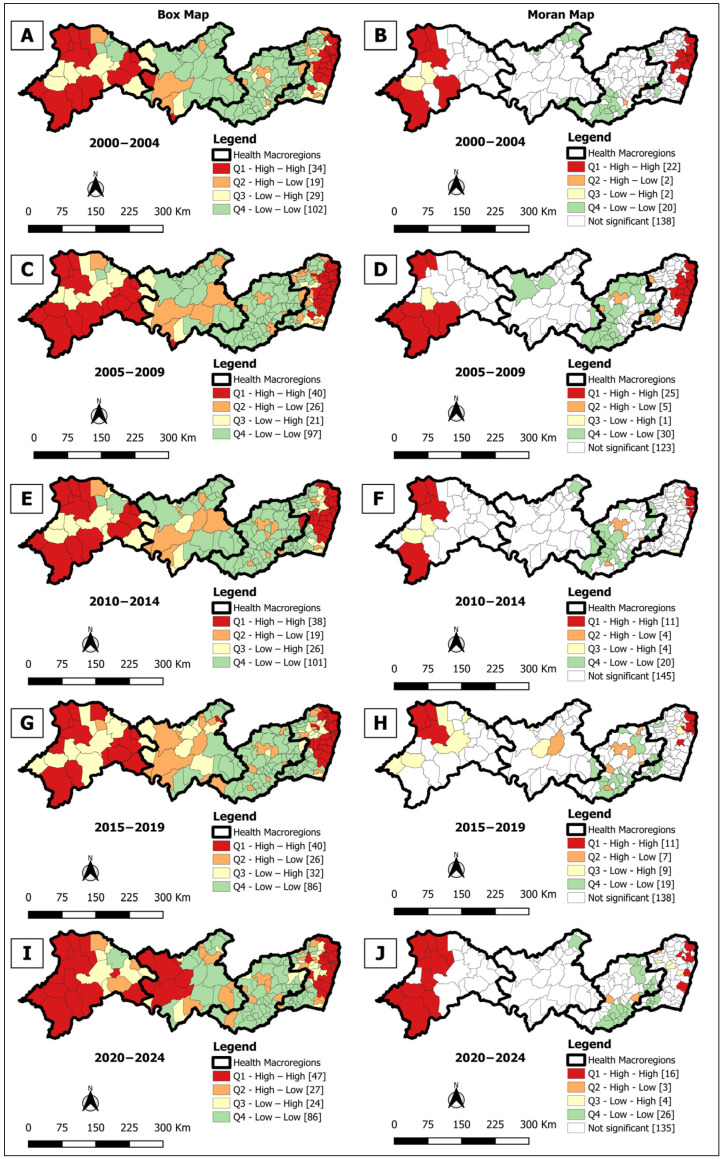
Spatial analysis on the general incidence rate of leprosy for Box Map (**A**,**C**,**E**,**G**,**I**) and Moran Map (**B**,**D**,**F**,**H**,**J**), per 100,000 inhabitants, according to the municipality. Pernambuco, Brazil, over five-year period (2000–2024).

The Box Map identified high-risk clusters (High/High) in all analyzed periods, with variations in distribution. There were 4 clusters in the first quinquennium (2000–2004) (Figure 3A), 3 in the second (2005–2009) (Figure 3C), and again 4 in the third (2010–2014) (Figure 3E) and fourth (2015–2019) (Figure 3G). In the last period (2020–2024) (Figure 3I), the number of municipalities in high-risk clusters increased to 47 (25.5%), concentrated mainly in macro-regions I, III, and IV, indicating greater territorial dispersion of the disease.

In turn, the Moran Map also evidenced high-risk clusters in all periods, concentrated especially in macro-regions I and IV. The number of priority municipalities varied from 22 (12.0%) in 2000–2004 to 25 (13.6%) in 2005–2009 (Figure 3B,D), dropping to 11 (6.0%) in 2010–2014 (Figure 3F) and remaining at 11 (6.0%) in 2015–2019 (Figure 3H) and increasing again in 2020–2024 to 16 (8.7%) (Figure 3J), indicating persistence of critical areas despite the reduction in crude detection rate.

### 3.2. Spatial Analysis of Incidence in Children Under 15 Years of Age

**Figure 4:**
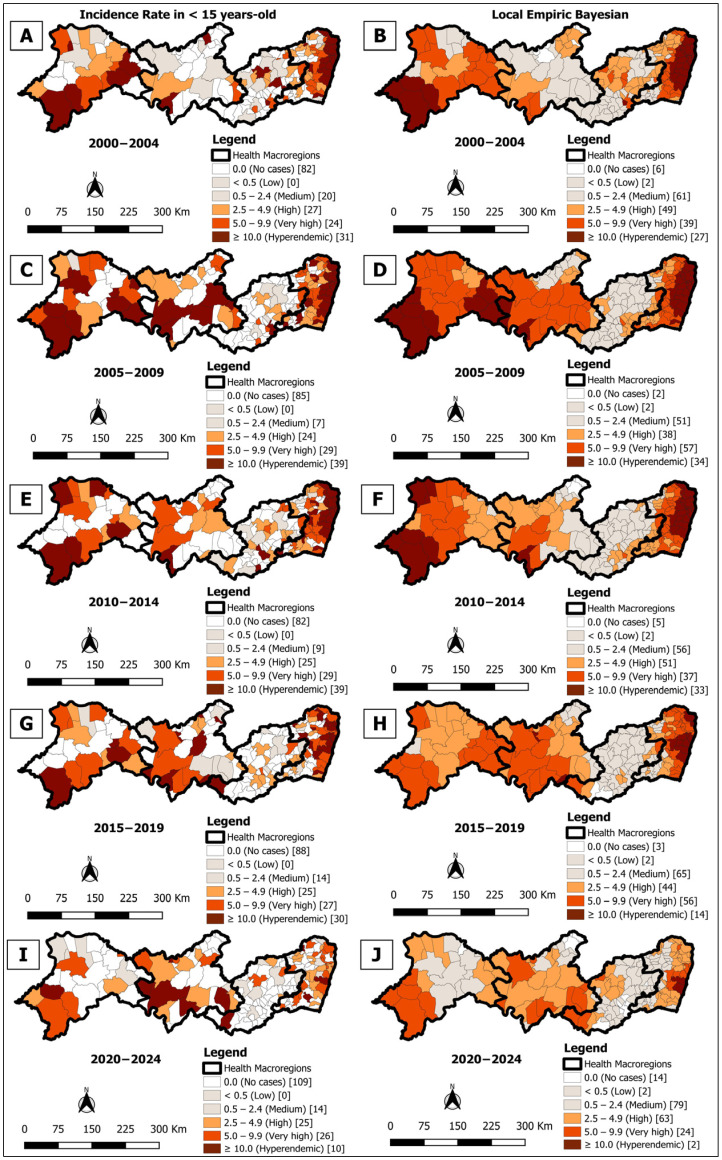
Spatial analysis on the general incidence rate of leprosy in the population aged from zero to 14 years: general incidence rate for individuals under 15 years of age (**A**,**C**,**E**,**G**,**I**) and rate smoothed using the local empirical Bayes method (**B**,**D**,**F**,**H**,**J**); per 100,000 inhabitants, according to the municipality. Pernambuco, Brazil, by five-year period (2000–2024).

In the analysis of the five quinquennia (2000–2024), crude detection rates in children under 15 years classified 31 (16.8%) municipalities as hyperendemic in 2000–2004 (Figure 4A), 39 (21.2%) in 2005–2009 (Figure 4C), 39 (21.2%) in 2010–2014 (Figure 4E), 30 (16.3%) in 2015–2019 (Figure 4G), and 10 (5.4%) in 2020–2024 (Figure 4I). Additionally, 24 (13.0%) municipalities presented very high endemicity in 2000–2004 (Figure 4A), 29 (15.8%) in 2005–2009 (Figure 4C), 29 (15.8%) in 2010–2014 (Figure 4E), 27 (14.7%) in 2015–2019 (Figure 4G), and 25 (13.6%) in 2020–2024 (Figure 4I).

Bayesian smoothing evidenced these areas with greater clarity, revealing 27 (21.2%) municipalities as hyperendemic in 2000–2004 (Figure 4B), 34 (18.5%) in 2005–2009 (Figure 4D), 33 (17.9%) in 2010–2014 (Figure 4F), 14 (7.6%) in 2015–2019 (Figure 4H), and 2 (1.1%) in 2020–2024 (Figure 4J), in addition to an expressive increase in the number of municipalities classified as very high endemicity in the quinquennia 2005–2009 with 57 (31.0%) (Figure 4D) and 2015–2019 with 56 (30.4%) (Figure 4H).

**Figure 5:**
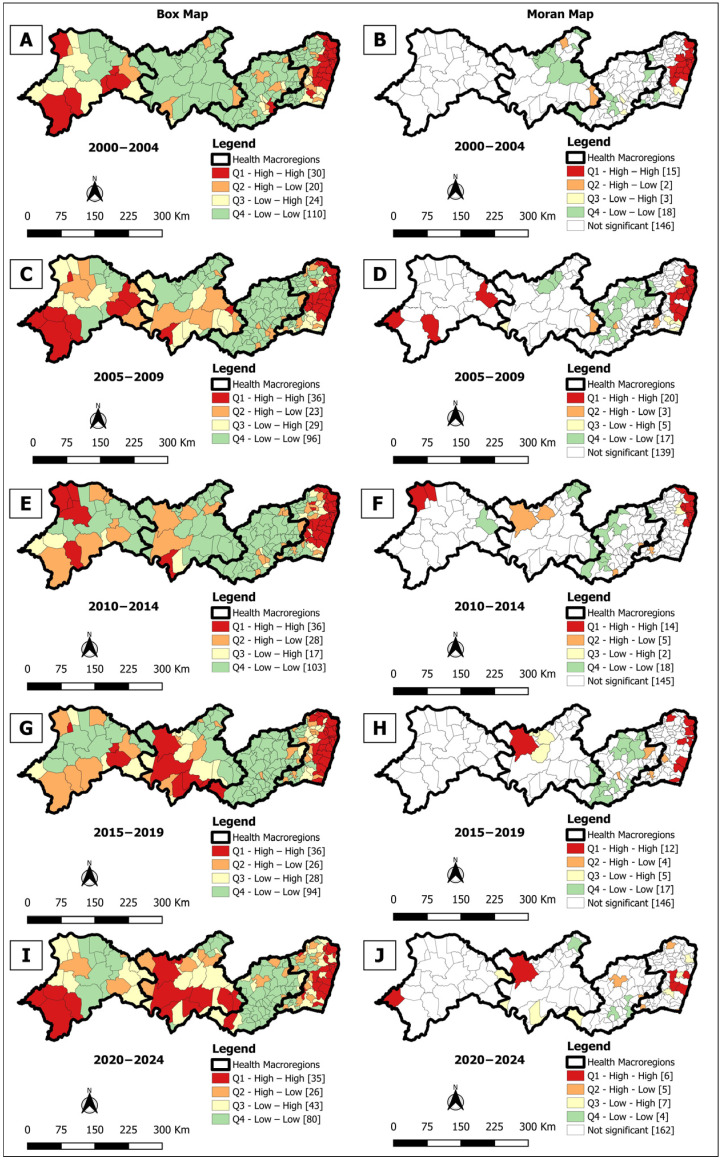
Spatial analysis on the general incidence rate of leprosy in the population aged from zero to 14 years: general incidence rate for Box Map (**A**,**C**,**E**,**G**,**I**) and Moran Map (**B**,**D**,**F**,**H**,**J**), per 100,000 inhabitants, according to the municipality. Pernambuco, Brazil, over five-year period (2000–2024).

The Box Map identified 30 (16.3%) priority municipalities in 2000–2004 (Figure 5A), 36 (19.6%) in the other three quinquennia (Figure 5C,E,G), and 35 (19.0%) in 2020–2024 (Figure 5I), with recurrent clusters in macro-region I and smaller groups in the other regions. In turn, the Moran Maps showed 15 (8.2%) priority municipalities in 2000–2004 (Figure 5B) located only in macro-region I, 20 (10.9%) in 2005–2009 (Figure 5D), 14 (7.6%) in 2010–2014 (Figure 5F), 11 (6.0%) in 2015–2019 (Figure 5H), and 5 (2.7%) in 2020–2024 (Figure 5J) concentrated mainly in macro-regions I and IV. In the last two quinquennia, one municipality was located in macro-region III (Figure 5H,J).

### 3.3. Spatial Analysis of the Incidence of Grade 2 Disability at Diagnosis

**Figure 6:**
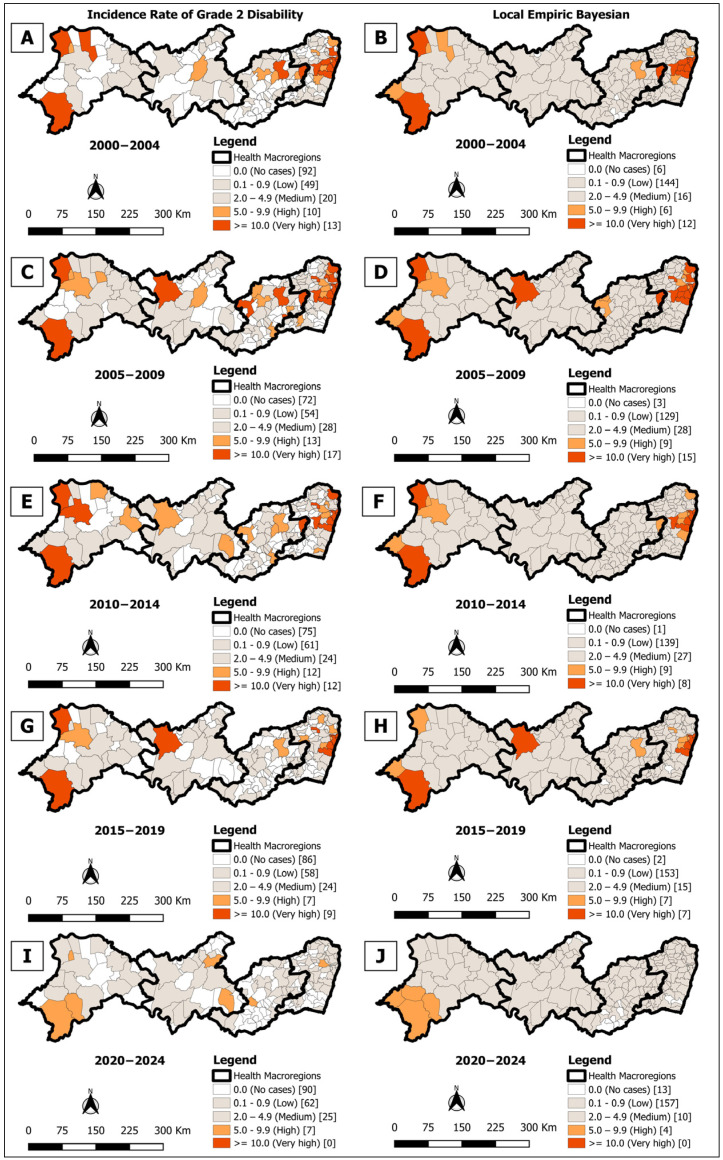
Spatial analysis on the rate of incidence of leprosy with a degree of physical disability at the time of diagnosis for incidence rate for cases with grade 2 disability (**A**,**C**,**E**,**G**,**I**) and rate smoothed using the local empirical Bayes method (**B**,**D**,**F**,**H**,**J**), per 100,000 inhabitants, according to the municipality. Pernambuco, Brazil, over five-year period (2000–2024).

In the analysis of the five quinquennia (2000–2024), a predominance of municipalities without records of grade 2 physical disability at diagnosis was observed, varying from 92 (50.0%) in 2000–2004 (Figure 6A) to 90 (48.9%) in 2020–2024 (Figure 6I). Among municipalities with occurrence, the majority concentrated in low and medium rate strata in all periods (Figure 6A,C,E,G,I). Bayesian smoothing revealed small groups with very high endemicity in all quinquennia, located mainly in macro-regions I and, to a lesser extent, in II, III, and IV (Figure 6B,D,F,H), with the exception of the last quinquennium (2020–2024) where the high cluster was located only in macro-region IV (Figure 6J).

**Figure 7:**
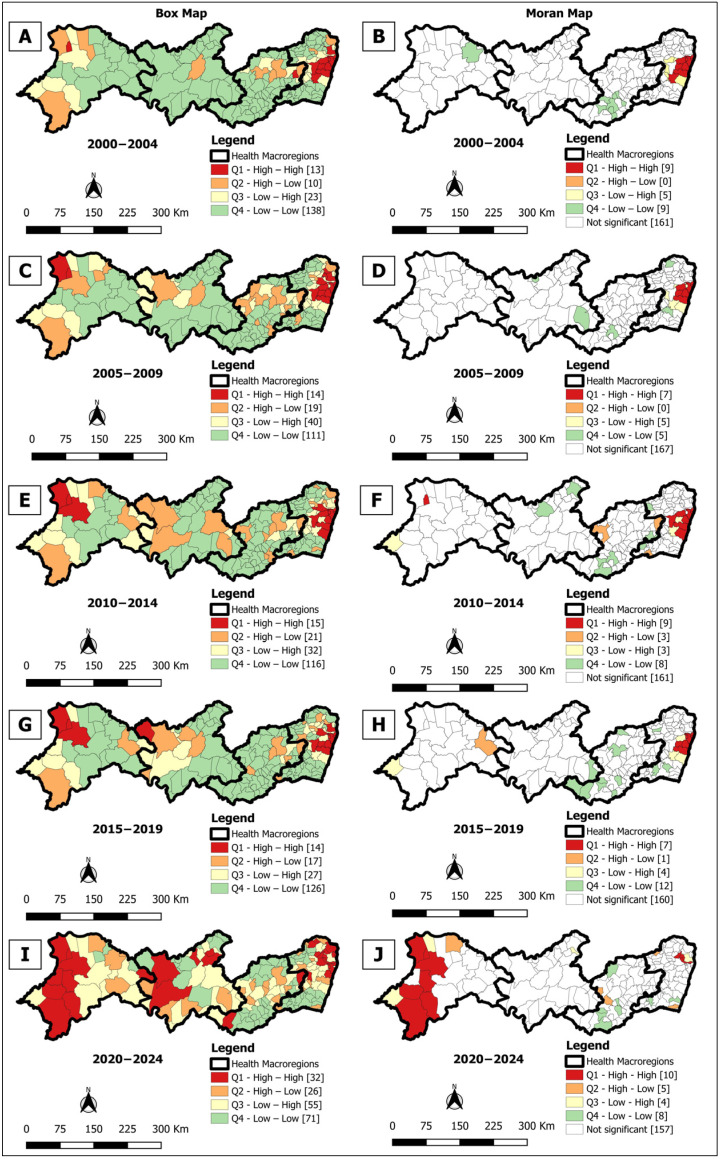
Spatial analysis on the rate of incidence of leprosy with a degree of physical disability at the time of diagnosis: incidence rate for Box Map (**A**,**C**,**E**,**G**,**I**) and Moran Map (**B**,**D**,**F**,**H**,**J**), per 100,000 inhabitants, according to the municipality. Pernambuco, Brazil, over five-year period (2000–2024).

The Box Maps highlighted recurrent high-risk clusters (High/High), especially in macro-region I, varying from 11 (6.0%) municipalities in 2000–2004 (Figure 7A) to 32 (17.4%) in 2020–2024 (Figure 7I), in addition to smaller groups in the other macro-regions (Figure 7A,C,E,G,I). Consistently, the Moran Maps confirmed the persistence of these clusters, located mainly in macro-region I (Figure 7B,D,F,H,J), with variation from 9 (4.9%) municipalities in the first quinquennium (Figure 7B) to 3 (1.6%) in the last (Figure 7J). In the quinquennia 2010–2014 and 2020–2024, municipalities were also found in macro-region IV, being one (0.5%) and seven (3.8%) respectively.

## 4. Discussion

The spatial analyses conducted showed significant spatial heterogeneity. Clusters of high overall detection rates of leprosy cases, active transmission, and late diagnosis were identified, mainly in macro-regions I and IV. The application of spatial statistics allowed the identification of critical areas of the disease in the state. While the maps of the gross average rates showed the general distribution, the thematic maps with the smoothed indicators, which attenuate the influence of small populations, revealed more precise spatial patterns [39]. Spatial dependency analysis was decisive in identifying priority areas for intervention [42], providing valuable information for improving the control of leprosy transmission.

Although the analysis showed a decline in detection rates and the persistence of clusters in specific macro-regions, it is important to consider the existence of silent areas, where low case detection does not necessarily indicate low leprosy occurrence. In these locations, operational factors such as diagnostic delays exceeding 10 months, presence of grade 2 disabilities at diagnosis, previous misdiagnoses, and excessive referrals may compromise early identification and mask the true magnitude of the disease [43]. Furthermore, the literature shows that areas with apparently low detection may, in fact, harbor active transmission foci, characterizing silent areas that require strengthened surveillance [44].

In the spatial distribution of the detection rate of new cases across the five-year periods analyzed, up to four clusters were observed in macro-regions I and IV. The persistence of areas of concentration of active leprosy transmission is associated with social determinants linked to poverty, such as low income, low educational level, precarious housing, inadequate sanitation, and limited access to health services [45,46]. These factors favor the circulation and persistence of the disease in the region [47,48].

The spatial pattern of occurrence observed in the Box Map for the detection rate in children under 15 was similar to the overall incidence indicator. In the analysis of areas with statistical significance (Moran Map), macro-region I stood out consistently in all five-year periods as an area requiring priority interventions. The detection rate in children under 15 reflects the active transmission of leprosy and the operational efficiency of the disease elimination program [49,50].

In the second, third and last five-year periods, the Moran Map analysis also identified municipalities in macro-region IV, corroborating the findings related to the overall detection rate. In the fourth and fifth five-year period, there was an indication of *Mycobacterium leprae* circulation within families or through extrafamilial contacts in macro-region III. This region has the lowest GDP in the state [34]. The study by Martoreli Júnior et al. [50] on the population under 15 years of age indicates that index cases usually reside in poorer and hyperendemic areas.

The spatial analysis of the gross average rates of grade 2 physical disability at the time of diagnosis revealed that, in all five-year periods, there was a predominance of municipalities with average, low or no case rates. These areas became more evident when applying the smoothed rates. The divergence between the gross and smoothed rate maps can be attributed to persistent operational problems in the municipalities. Souza et al. [51] list these problems as underreporting, errors in data entry, low coverage of health teams, lack of professionals trained in diagnosis, poor follow-up of patients and frailties in the surveillance sectors. Moreover, the COVID-19 pandemic significantly impacted global health, leading to the suspension of many routine health services and thereby hindering the timely diagnosis of other infections, including leprosy [52].

The Box Maps for grade 2 physical disability at diagnosis revealed clusters similar to those observed in the general incidence rates and in children under the age of 15. In the Moran maps, a significant cluster was identified in macro-region I in all five-year periods, suggesting a constant hidden prevalence and delays in diagnosis [53,54]. This indicator is crucial for assessing timely detection, since early diagnosis of leprosy can be challenging due to the similarity of symptoms with other skin diseases and neuropathic problems [55,56].

Grade 2 physical disability at diagnosis is an indicator of the quality of health services [56]. Although macro-region I has 92.4% coverage by family health teams, difficulties still persist in the timely detection of new cases [34]. The Family Health Strategy Program teams play a fundamental role in reorganizing care for leprosy patients, ensuring access, diagnosis and treatment [57]. However, the data presented highlights the need for a differentiated approach to leprosy diagnosis in macro-region I.

This research recognizes the possible limitations arising from the use of secondary data, which may present inconsistencies in the filling in and processing of records. However, in order to mitigate these limitations, duplicates, incompleteness and inconsistencies in the notification forms were identified and excluded. It should be emphasized that SINAN database is the only system available that integrates epidemiological data on leprosy.

Priority areas for intervention were identified in macro-regions I and IV, with evidence of *Mycobacterium leprae* circulation in macro-region III. This situation is the result of a combination of factors, including weaknesses in the decentralization of health services in Primary Health Care, population difficulties in accessing the services, limitations in the capacity of services to diagnose new cases, and failures in surveillance systems. Further studies are needed to better understand these associations, and research into the leprosy program in the state is therefore recommended, focusing on operational difficulties, especially in early diagnosis.

## 5. Conclusions

The spatial pattern of leprosy was heterogeneous, with clusters of high overall detection rates, associated with active transmission and late diagnosis, mainly located in macro-regions I (Metropolitana) and IV (Vale do São Francisco and Araripe). The spatial agglomeration in macro-region III (Sertão) revealed that the diagnosis of new cases also occurred late in this area.

Therefore, considering the current leprosy scenario in the state and the pledges proposed by the WHO and the Ministry of Health to eliminate the disease, this study can serve as a guide for action and policy implementation. It is necessary to combat challenges that go beyond the health sector, including demographic, socio-economic and environmental factors that influence the transmission of the disease.

The results of this study can contribute to the evaluation of the leprosy program, helping in the process of planning, monitoring and, consequently, making decisions to tackle leprosy as a public health problem. This, in turn, will have an impact on the quality of health services provided to patients affected by the disease.

## Figures and Tables

**Table 1:** Sociodemographic and economic characterization of the health macro-regions of Pernambuco, Brazil, 2019.

Sociodemographic and Economic Variables	Pernambuco	Health Macro-Regions			
		I	II	III	IV
Total population (inhabitants)	9,557,070	5,771,869	1,922,110	855,144	1,007,947
Territorial area (Km^2^)	98,068	13,607.7	18,578.3	30,262.6	35,619.5
Demographic density (hab./Km^2^)	97.5	424.2	103.5	28.3	28.3
% Households with water supply (general water supply network)	76.0	80.1	70.7	59.9	73.0
% Households by sanitary installation (rainwater network)	43.7	38.8	56.1	44.5	47.5
% Households by type of waste disposal (Collected)	81.6	88.3	77.3	58.5	67.3
GDP per capita (R$)	19,164.50	15,852.30	10,387.40	9258.60	9382.70
GDP Current values (R$) (Million)	181,550,642.01	135,260,101.18	24,890,956.66	8,989,749.64	12,409,834.54
Life expectancy at birth	71.1	70.9	69.7	69.9	70.6
Human Development Index (HDI) (average)	0.673	0.614	0.574	0.593	0.598
% Literacy	83.3	77.3	71.4	75.3	77.4
Population National Health System (SUS) dependent (inhabitants)	8,168,411	4,495,281	1,788,020	831,992	1,053,214
% population SUS-dependent	86.0	80.5	93.7	97.9	91.2
% Population coverage estimated by the Community Health Agent Teams	84.6	94.9	93.7	98.6	98.6
% Estimated population coverage by Health Teams	80.6	92.4	96.2	94.6	90.6

Source: Pernambuco State Health Department (2021) [34].

**Table 2:** Epidemiological indicators of leprosy for spatial analysis.

Indicator	Calculation Method	Usefulness	Parameters
Average annual incidence of leprosy per 100,000 inhabitants	Numerator: new cases resident in a given location per five-year period. Divided by 5. Denominator: resident population of the middle year of each five-year period in the same location. Multiplication factor: 100,000	To measure morbidity strength, magnitude, and endemic trend.	Hyperendemic: ≥ 40.0/100,000 inhabitants Very high: 20.0 to 39.9/100,000 inhabitants High: 10.0 to 19.9/100,000 inhabitants Medium: 2.0 to 9.9/100,000 inhabitants Low: <2.0/100,000 inhabitants
Average annual incidence of leprosy among individuals aged zero to 14 years per 100,000 inhabitants	Numerator: new cases in children under 15 years of age, resident in a given location per five-year period. Divided by 5. Denominator: resident population aged 0 to 14 of the middle year of each five-year period in the same place. Multiplication factor: 100,000	To measure the strength of recent transmission of the endemic disease and its trend.	Hyperendemic: ≥ 10.0/100,000 inhabitants Very high: 5.0 to 9.9/100,000 inhabitants High: 2.5 to 4.9/100,000 inhabitants Medium: 0.5 to 2.5/100,000 inhabitants. Low: <0.5/100,000 inhabitants
Average rate of incidence of grade 2 physical disability at the time of diagnosis per 100,000 inhabitants	Numerator: new cases with grade 2 physical disability at diagnosis, resident in a given location per five-year period. Divided by 5. Denominator: resident population of the middle year of each five-year period in the same place. Multiplication factor: 100,000	To evaluate deformities caused by leprosy in the general population and the monitoring of the trend of timely detection of new cases.	Very high: ≥10.0/100,000 inhabitants. High: 5.0 to 9.9/100,000 inhabitants. Medium: 2.0 to 4.9/100,000 inhabitants. Low: 0.1 to 1.9/100,000 inhabitants.

Source: Brazil (2023) [37]; Monteiro et al. (2015) [38].

**Table 3:** Moran’s statistic for epidemiological indicators. Pernambuco, Brazil, 2000–2024.

Five-Year Period	Overall Detection Rate		Detection Rate in Children Under 15 Years of Age		Detection Rate with Grade 2 Physical Disability at the Time of Diagnosis	
	Moran’s I	*p* Value	Moran’s I	*p* Value	Moran’s I	*p* Value
2000–2004	0.3	0.01	0.2	0.01	0.4	0.01
2005–2009	0.4	0.01	0.3	0.01	0.3	0.01
2010–2014	0.3	0.01	0.4	0.01	0.3	0.01
2015–2019	0.3	0.01	0.1	0.01	0.3	0.01
2020–2024	0.3	0.00	0.0	0.04	0.1	0.00

## Data Availability

The original datasets used in this study contain nominal information and were accessed under authorization from the State Health Department of Pernambuco, following approval by the Ethics Committee of the Aggeu Magalhães Institute (IAM)/Oswaldo Cruz Foundation (approval number 5.245.542). To ensure compliance with the Brazilian General Data Protection Law (Lei Geral de Proteção de Dados Pessoais–LGPD, Law No. 13.709/2018), these microdata cannot be publicly shared. However, an anonymized and aggregated dataset has been prepared and is provided as Supplementary Material. This supplementary dataset is fully anonymized and allows replication of the analyses presented in this study.

## Supplementary Materials

The following supporting information can be downloaded at: https://www.mdpi.com/article/10.3390/tropicalmed10120327/s1, Table S1: Database of epidemiological indicators and mid-period population estimates for each five-year interval by municipality of residence.
